# Tetanus – a lethal and neglected infectious disease

**DOI:** 10.1097/MS9.0000000000003407

**Published:** 2025-05-20

**Authors:** Priya Karna, Amit Kumar Thakur

**Affiliations:** Manipal College of Medical Sciences, Manipal, Nepal

**Keywords:** case report, intravenous immunoglobulin (IVIG), tetanus, vaccination

## Abstract

**Introduction and importance:**

Tetanus, caused by *Clostridium tetani*’s toxin, remains a deadly disease, particularly in tropical regions. Generalized tetanus, the most severe form, leads to painful muscle spasms due to tetanospasmin binding to motor neurons. Early diagnosis and treatment are crucial, especially in developing countries with limited vaccination access, highlighting the importance of routine immunization and addressing healthcare challenges in low-resource settings.

**Case presentation:**

A 55-year-old male with a history of alcohol abuse presented with classic tetanus symptoms, including muscle rigidity, trismus, and opisthotonus. Despite intravenous medications and antibiotics, his condition worsened, requiring intubation, mechanical ventilation, and resuscitation for cardiac complications. Unfortunately, due to financial constraints, he left the ICU against medical advice and died shortly after discharge.

**Clinical discussion:**

The incidence of tetanus has significantly decreased in affluent nations since the 1940s due to widespread vaccination. From 2009 to 2017, 264 cases were reported in the U.S. Despite global efforts, tetanus remains a public health concern, with the World Health Organization reporting 21,897 cases globally in 2023, reflecting ongoing gaps in prevention, vaccination coverage, and healthcare access. Generalized tetanus, characterized by painful muscle spasms and autonomic dysfunction, is more common than localized or cephalic tetanus. Diagnosis is clinical, and treatment involves wound care, managing spasms, administering anti-tetanus immunoglobulin, and an accelerated immunization regimen for long-term protection.

**Conclusion:**

This case highlights the critical need for vigilance and vaccination programs in areas with limited healthcare resources. Tetanus continues to be a major public health concern, and timely interventions, such as intravenous immunoglobulin, can be lifesaving. In developing countries, prioritizing routine vaccination and booster shots is essential to reduce the devastating impact of this preventable disease.

## Introduction

Tetanus is a severe and potentially fatal infectious disease that can have serious consequences if not promptly identified and treated. This infection is more commonly observed in tropical regions^[1]^. Tetanus is an illness triggered by the toxin generated by the bacterium *Clostridium tetani*^[2]^. *C. tetani* is an anaerobic bacterium, gram-positive in nature, and capable of forming spores. It is commonly present in soils worldwide, as well as in the intestinal tracts and excrements of various animals. The primary virulence factor of *C. tetani* is the tetanus toxin, also known as tetanospasmin, which has a significant impact on motor neurons, leading to tetanic convulsions^[3]^.

**HIGHLIGHTS**

- A 55-year-old male presented with severe muscle spasms, trismus, and opisthotonus, diagnosed as generalized tetanus.
- History included alcohol abuse and a fall injury, but no recent wound was identified.
- Treated with antibiotics, diazepam, and intravenous immunoglobulin (IVIG), with mechanical ventilation for severe spasms.
- Complications included cardiac asystole requiring resuscitation.
- Patient left ICU due to financial constraints and succumbed shortly after discharge.
- Case emphasizes the importance of vaccination and challenges in managing tetanus in resource-limited settings.

Tetanus can manifest in four distinct clinical patterns: generalized, localized, cephalic, and neonatal. The entry of tetanus spores into the body occurs through breaches in the skin, and these spores then undergo germination to generate a neurotoxin known as tetanospasmin, responsible for the spasms characteristic of tetanus^[4]^. Tetanospasmin inhibits the release of neurotransmitters that have an inhibitory effect, such as GABA and glycine, leading to intense and painful muscle contractions^[5]^. Neurotransmitters like GABA and glycine play a role in inhibiting motor neurons. Lack of central inhibition can lead to hyperactivity, uncontrolled muscle spasms, and spastic paralysis. The toxin binds irreversibly to neurons, and restoration of neural function occurs through the sprouting of new nerve terminals and the formation of new synapses^[6]^.

## Case report

A 55-year-old male presented to the emergency department with complaints of backache, severe enough to restrict daily activities, diffuse, dull aching, and generalized muscle spasm with rigidity and facial spasm for 1 day. The patient experienced a tingling sensation throughout his body. He had a history of fall injury 3 years ago, for which he was admitted to the hospital. The patient did not recall any recently infected wounds. The patient consumed local homemade alcohol of about 500–600 mL five to six times per week. He also had a smoking history of approximately five to six cigarettes per day for 10 years. There was no significant medical or surgical history. He had no known allergies.

On presentation, he appeared to have borderline hypertension with a blood pressure of 140/100 mm Hg, pulse rate of 110 beats/min, respiratory rate of 22/min, temperature of 98.6 °F, and oxygen saturation of 92% in room air.

Examination revealed his Glasgow Coma Scale (GCS) of spontaneous eye-opening (E), oriented verbal response (V), and obeying the command on motor response (M). He was conscious and well-oriented to time, place, and person; his memory was intact and his speech was normal. However, neck rigidity and trismus were also observed. His pupils were 4 mm bilaterally and reactive to light. The results of the spatula test were positive. The gag reflex was not observed. Muscle bulk and reflexes were normal with a power of 5/5 in the bilateral upper and lower extremities, but there was a diffuse increase in muscle tone in all extremities. Bilateral plantar reflexes were downgoing. On auscultation, breath sounds were mildly reduced bilaterally over the chest.

Laboratory findings were as follows: leukocytes, 12.47 /mm^3^ (normal: 4–11/mm^3^); hemoglobin, 16 g/dL (normal: 13.5–17.5 g/dL); and platelets, 332/mm^3^ (normal: 150–450/mm^3^). In the biochemical findings, his total calcium was 10.3 mg/dL (normal: 8.5–10.5 mg/dL), sodium 146 mmol/L (normal: 135–145 mmol/L), potassium 4.1 mmol/L (normal: 3.5–4.5 mmol/L), creatinine 1.2 mg/dL (normal: 0.6–1.2 mg/dL), urea 37 mg/dL (normal: 7–20 mg/dL), and creatinine kinase 205 U/L (normal: 40–250 U/L.

He initially received intramuscular diclofenac 75 mg, tab. clonazepam 0.25 mg orally, intravenous diazepam 5 mg, and intravenous ceftriaxone 1 g in the emergency room, and was admitted to the intensive care unit, with differential diagnosis of acute meningitis, tetanus, strychnine poisoning with modification of antibiotics to ceftriaxone and metronidazole. The patient was afebrile and had no episodes of vomiting or seizures during the hospital stay.

On day 2, he developed a locked jaw and risus sardonicus; his muscle contraction was excessive with no relaxation in between. It was progressive, and he was in extreme pain. He received intravenous diazepam in infusion and pantoprazole. He was kept in a dark isolated room with a provisional diagnosis of tetanus. He also received tetanus intravenous immunoglobulin (IVIG). A lumbar puncture could not be performed because of the stiff back, and a CT scan of the head was reported to be normal. Despite sedation with intravenous diazepam, muscle spasms increased, and occasional severe generalized spasms were noted leading to an opisthotonus position.

On day 3, the patient was intubated and maintained on mechanical ventilation in anticipation of airway obstruction from laryngeal and pharyngeal spasms. He received intravenous midazolam and vecuronium infusions to relieve the pain and spasms. On day 7, the patient went on asystole, for which cardiopulmonary resuscitation was performed, and he received three cycles of atropine with two cycles of adrenaline that restored the normal rhythm of the heart. Calcium gluconate was administered to the patient. The progression of GCS of the patient over 7 days of hospital stay is mentioned in Table 1.

**Table 1:** Progression of GCS over 7 days of hospital stay

Days	1	2	3	4	5	6	7
GCS	15/15	7/15	3/15	3/15	3/15	3/15	3/15

The patient’s family was counseled about the course of the illness and the cost of treatment. Due to financial difficulties, the patient left the ICU against medical advice on the eighth day of admission and succumbed to the disease a day after discharge.

**Figure 1.**
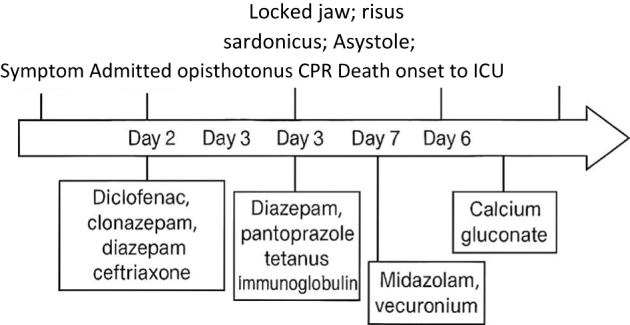
Timeline diagram illustrating the sequence of symptom onset, treatment interventions, and clinical progression in a 55-year-old male with tetanus.

The timeline illustrating the sequence of symptom onset, treatment interventions, and clinical progression is mentioned in Fig. 1.

## Discussion

The occurrence of tetanus has significantly decreased since the 1940s owing to widespread tetanus toxoid vaccination programs in affluent nations. According to the United States Centers for Disease Control and Prevention, there were a total of 264 tetanus cases reported between 2009 and 2017^[7]^. According to the World Health Organization, the number of reported global tetanus cases has varied in recent years, with 14 745 cases in 2019, 11 897 in 2020, 9828 in 2021, 6705 in 2022, and a notable increase to 21 897 cases in 2023, indicating fluctuations that may reflect differences in surveillance, reporting accuracy, and access to immunization programs^[8]^.

Tetanus remains a significant concern among adults in numerous low- and middle-income countries, primarily because of the absence or interruption of vaccination initiatives^[9]^. Generalized tetanus is considerably more prevalent than the localized form, characterized by painful muscle spasms near the wound site, often progressing to the former type. Cephalic tetanus, a less common subtype of localized tetanus, is characterized by symptoms such as difficulty in swallowing (dysphagia), lockjaw (trismus), retracted eyelids, deviated gaze, and a sardonic grin (risus sardonicus), primarily affecting the bulbar musculature^[10]^. Generalized tetanus is notable for its autonomic involvement, leading to rapid fluctuations in blood pressure and heart rate, which can pose considerable management challenges^[11]^.

The diagnosis of tetanus relies solely on clinical evaluation^[11]^. As seen in this case, clinical examination and neurological examination findings were vital in diagnosing tetanus.

Effective management of generalized tetanus involves measures such as preventing further toxin absorption, managing muscle spasms, cleaning and debriding wounds, and providing supportive care. The widely accepted treatment for neutralizing tetanospasmin is the administration of human anti-tetanus immunoglobulins^[12]^. The second and crucial step in managing this condition involves administering tetanus immunoglobulin, a measure that significantly reduces mortality associated with generalized tetanus. Typically, it is advisable to provide a tetanus accelerated immunization regimen, including immunization upon presentation or in high-risk situations, at discharge, and again 4 weeks later. This approach ensures robust immunity and minimizes future risks^[13]^.

Our patient’s health worsened even after the proper clinical therapy was started, which included sedation, IVIG, antibiotics, and mechanical breathing. Limited availability to early immunoglobulin therapy and delayed diagnosis as a result of unusual presentation could be contributing factors. Most notably, critical care was interrupted when financial limitations resulted in release against medical advice. This instance demonstrates how, even with treatment based on guidelines, socioeconomic constraints, and systemic healthcare limits can harm outcomes.

## Conclusion

Tetanus is still observed in some developing countries, with a lack of proper vaccination and poor socioeconomic status. It is strictly a clinical diagnosis and one should have a high index of suspicion, particularly in the developing world. Tetanus is a vaccine-preventable disease, the focus of which is the routine vaccination of children and women of childbearing age and regular booster immunization of the general population. Although the prognosis is poor in generalized tetanus, conservative management with intensive care unit admission and IVIG can be helpful.

## Research registration unique identifying number (UIN)

Not done.

## Method

Work has been reported in line with the SCARE criteria^[14]^.

## References

[1] Aboyans V. Causes of Death Collaborators. Global, regional, and national age-sex specific all-cause and cause-specific mortality for 240 causes of death, 1990-2013: a systematic analysis for the Global Burden of Disease Study 2013. Lancet (British Edition) 2015;385:117–71. 10.1016/S0140-6736(14)61682-2 PMC4340604 25530442

[2] Raia PJ. Tetanus: a case study. J Am Board Fam Med 2001;14:223–24. 11355056

[3] Dolin R Bennett JE Mandell GL. Mandell, Douglas, and Bennett’s principles and practice of infectious diseases. In Mandell, Douglas, and Bennett’s Principles and Practice of Infectious Diseases; 2005:1727–3662.

[4] Centers for Disease Control, Prevention (US), National Immunization Program (Centers for Disease Control, Prevention), National Immunization Program (Centers for Disease Control, Prevention). Education, Information, Partnership Branch. Epidemiology and prevention of vaccine-preventable diseases. Department of Health and Human Services, Public Health Service, Centers for Disease Control and Prevention; 2005:1727–3662.

[5] Tintinalli J Kelen GD Stapczynski JS. Emergency Medicine, Comprehensive Study Guide. American College of Physicians. The McGraw-Hill Companies, Inc. Sixth Edition, Section 2004;22:1545–46.

[6] Masuyer G Conrad J Stenmark P. The structure of the tetanus toxin reveals pH-mediated domain dynamics. EMBO Rep 2017;18:1306–17. 28645943 10.15252/embr.201744198 PMC5538627

[7] US Department of Health and Human Services, Centers for Disease Control and Prevention. Tetanus. Atlanta, GA: CDC; 2020.

[8] World Health Organization. Tetanus reported cases and incidence. Geneva: WHO; 2024 [cited 2025 Apr 10]. Available from: https://immunizationdata.who.int/global/wiise-detail-page/tetanus-reported-cases-and-incidence?CODE=Global&DISEASE=TTETANUS

[9] Tosun S Batirel A Oluk AI. Tetanus in adults: results of the multicenter ID-IRI study. Eur J Clin Microbiol Infect Dis 2017;36:1455–62. 28353183 10.1007/s10096-017-2954-3

[10] Gupta V Dewangan S Dev Bhatia B. Localised tetanus: a rare presentation of a ‘forgotten’ disease. J Paediatr Child Health 2011;47:152. 21401777 10.1111/j.1440-1754.2011.02007.x

[11] Sah SP Khanal S Dahal S. Generalized tetanus in an elderly patient: a case report. Ann Med Surg 2022;81:104465. 10.1016/j.amsu.2022.104465 PMC9486737 36147146

[12] De Barros Miranda-filho D de Alencar Ximenes Ra Barone AA. Randomized controlled trial of tetanus treatment with antitetanus immunoglobulin by the intrathecal or intramuscular route. BMJ 2004;328:615. 15003976 10.1136/bmj.38027.560347.7C PMC381133

[13] Gulamhussein MA Li Y Guha A. Localized tetanus in an adult patient: case report. J Orthop Case Rep 2016;6:100. 10.13107/jocr.2250-0685.592 PMC5288609 28164065

[14] Sohrabi C Mathew G Maria N. The SCARE 2023 guideline: updating consensus Surgical Case Report (SCARE) guidelines. Int J Surg 2023;109:1136–40. 37013953 10.1097/JS9.0000000000000373 PMC10389401

